# Selective Hydrogenation of Azobenzene to Hydrazobenzene via Proton-Coupled Electron Transfer from a Polyoxotungstate Cluster

**DOI:** 10.1021/jacsau.4c00127

**Published:** 2024-03-21

**Authors:** Zhou Lu, Shannon E. Cooney, James R. McKone, Ellen M. Matson

**Affiliations:** † Department of Chemistry, University of Rochester, Rochester, New York 14627, United States; ‡ Departments of Chemical and Petroleum Engineering and Chemistry, University of Pittsburgh, Pittsburgh, Pennsylvania 15260, United States

**Keywords:** Proton-coupled electron transfer, polyoxotungstate, electrochemistry, bond dissociation free energy, semihydrogenation

## Abstract

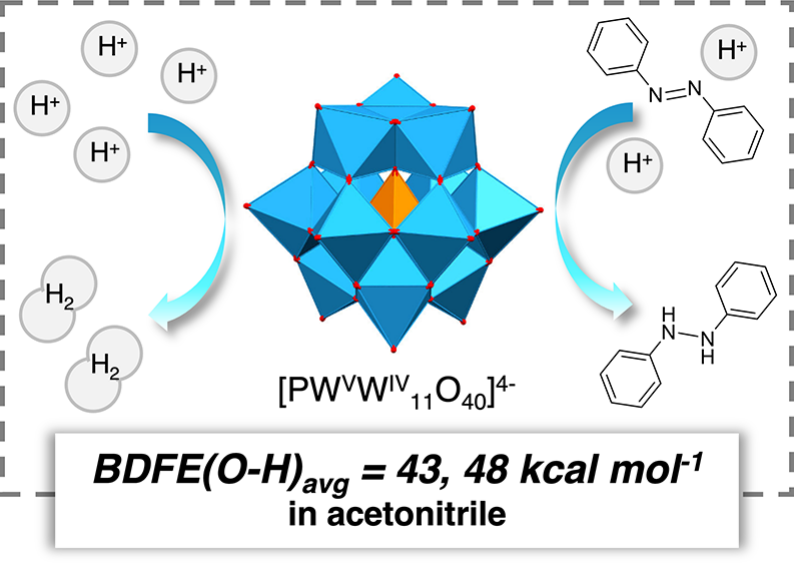

In this report, we describe proton-coupled electron transfer (PCET) reactivity at the surface of the Keggin-type polyoxotungstate cluster [^*n*^Bu_4_N]_3_[PW^VI^_12_O_40_] (**PW**_**12**_) in acetonitrile. Bond dissociation free energies (BDFEs) of the O–H groups generated upon reduction of **PW**_**12**_ in the presence of acid are determined through the construction of a potential–p*K*_a_ diagram. The surface O–H bonds are found to be weak (BDFE(O–H)_avg_ < 48 kcal mol^–1^), comparable to the BDFE of H_2_. This is consistent with the observed formation of H_2_ upon addition of a suitably strong organic acid, H_2_NPh_2_^+^ (p*K*_a MeCN_ = 5.98), to the reduced form of the cluster. The one-electron reduced form of **PW**_**12**_ is isolated and used in conjunction with acid to realize the stoichiometric semihydrogenation of azobenzene via PCET from the surface of the reduced cluster.

Hydrogenation is a fundamental transformation in the chemical industry. Prominent examples of catalysts that facilitate hydrogenation reactions include noble metal complexes (e.g., Ru, Ir, Pd, etc.),^1−4^ frustrated Lewis pairs,^5,6^ and extended metal surfaces (e.g., Raney nickel and Pd/C).^7,8^ While ubiquitous, these catalysts are often expensive, or otherwise produce stoichiometric byproducts that are challenging to recycle.^9,10^ Furthermore, high-pressure H_2_ gas produced from methane cracking is often used—this process requires large energy inputs and possesses a large carbon footprint.^11^ Accordingly, the development of inexpensive hydrogenation catalysts that facilitate selective small molecule reductions through H atoms sourced from nonfossil feedstocks remains an important objective.

A strategy to bypass the technological and economic limitations of H_2_ is the use of protons and chemical or electrochemical reducing equivalents as the hydrogen source.^12,13^ This reactivity is often conceptualized as proton-coupled electron transfer (PCET).^14−16^ One class of materials that exhibits PCET reactivity is reducible metal oxides (e.g., ceria, NiO, ZnO, and TiO_2_). In their reduced forms, these materials possess surface O–H bonds that are capable of mediating H atom transfer to small molecule substrates.^17−19^ To date, the fundamental mechanisms of PCET at metal oxide surfaces remain the subject of considerable debate.^20−22^ Extended metal oxides can insert H atoms, presenting the challenge in mechanism assignment that possible reactivity originates from the bulk versus being mediated surface-bound H atom equivalents. Similarly, extended metal oxide materials possess a wide range of surface terminations, leaving open the possibility that a small minority of surface sites (e.g., defects) are responsible for the observed reactivity. This ambiguity ultimately impairs the rational design of oxide-based H-transfer catalysts.^23−25^

To address these gaps in knowledge, our research team is investigating the reactivity of molecular tungsten oxide assemblies as atomically precise models for H atom uptake and transfer at the surfaces of extended tungsten oxide materials (Figure 1a). Polyoxotungstates (POTs) are soluble cluster complexes with multiple redox-active tungsten oxyanions linked together by bridging oxide units (Figure 1b). The surface structure of POTs is reminiscent of extended tungsten oxides—both are composed of W^VI^O_6_ octahedra bound together with bridging oxygens.

**Figure 1:**
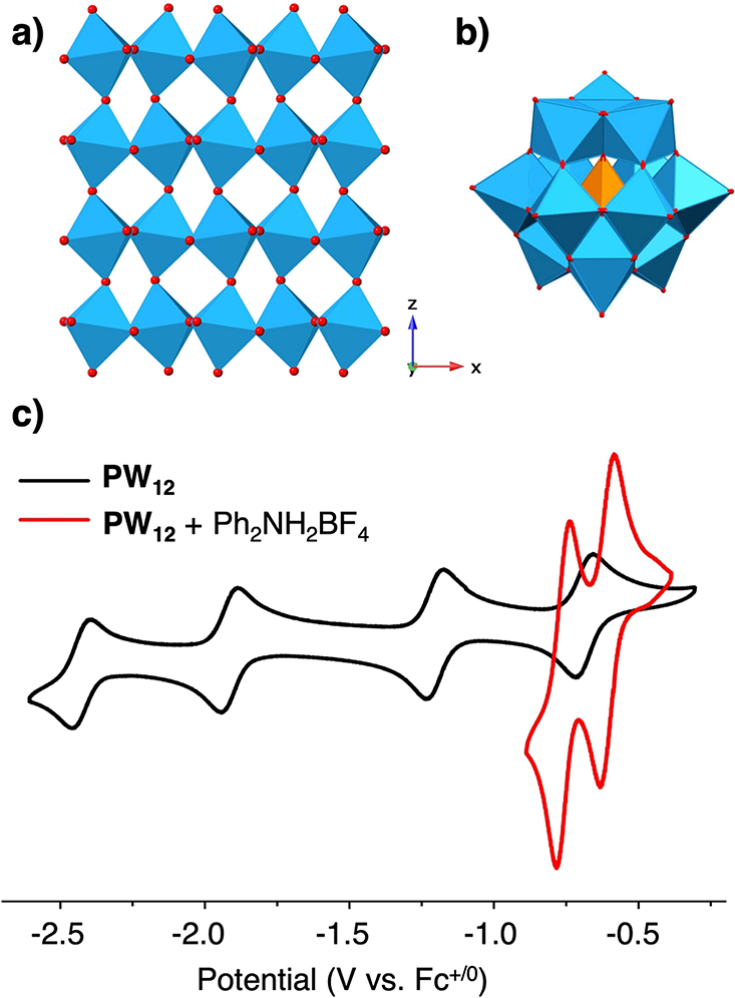
(a) Molecular structure of WO_3_ (monoclinic form stable at 25 °C). (b) Molecular structure of **PW**_**12**_. (c) Cyclic voltammogram of **PW**_**12**_ obtained in acetonitrile (black, 100 mM ^*n*^Bu_4_NPF_6_ with a scan rate of 200 mV/s) and in the presence of 4 equiv of Ph_2_NH_2_BF_4_ (p*K*_a_ = 5.98; red, 100 nM ^*n*^Bu_4_NPF_6_ with a scan rate of 200 mV/s).

The electrochemical properties of POTs depend strongly on the identity and chemical activities of cations in solution.^26^ For example, the Keggin-type POT cluster [^*n*^Bu_4_N]_3_[PW^VI^_12_O_40_] (**PW**_**12**_), in acetonitrile exhibits four reversible 1e^–^ redox events (Figure 1c) in aprotic electrolytes. However, upon addition of an excess of acid, these processes collapse into two positively shifted multielectron redox events. This behavior is broadly consistent with PCET, a hallmark of which entails reduction potentials that shift with the chemical activity of protons in solution. This observation suggests that reduced and protonated forms of **PW**_**12**_ possess reactive H atom equivalents at their surfaces.^27^ Unfortunately, the understanding of the thermodynamics of surface hydroxides at reduced POT clusters is limited by the challenge of isolating protonated forms of electron-rich polyoxometalates. These clusters are thought to be prone to disproportion reactions or hydrogen evolution as a result of exceptionally weak O–H bonds.^28,29^

To circumvent challenges measuring the BDFE(O–H) values of isolated, reduced forms of **PW**_**12**_, we used electrochemical measurements to map out the stable compositions using a potential–p*K*_a_ diagram.^30^ The electrochemical profile of **PW**_**12**_ in acetonitrile in the presence of organic acids with p*K*_a_ values ranging from 5 to 38 was assessed via cyclic voltammetry (CV; Figure 2, Figure S1, see the SI for additional details). The observed shifts in reduction potentials (*E°*) of **PW**_**12**_ in the presence of the selected range of organic acids indicate that electron transfer to the cluster is coupled to proton transfer under appropriate conditions (Figure 2b). The slopes of each multielectron event (55.5, 57.5 mV/p*K*_a_ unit) are near the theoretical value of 59 mV/p*K*_a_ unit for an n-electron, n-proton transfer. Locating the intersections of acid dependent and independent regions yields acid dissociation constants of 34.8 ± 0.6 (p*K*_a_1, eq S1), 25.9 ± 0.4 (p*K*_a_2, eq S2), 17.4 ± 0.4 (p*K*_a_3, eq S3), and 8.3 ± 0.2 (p*K*_a_4, eq S4).

**Figure 2:**
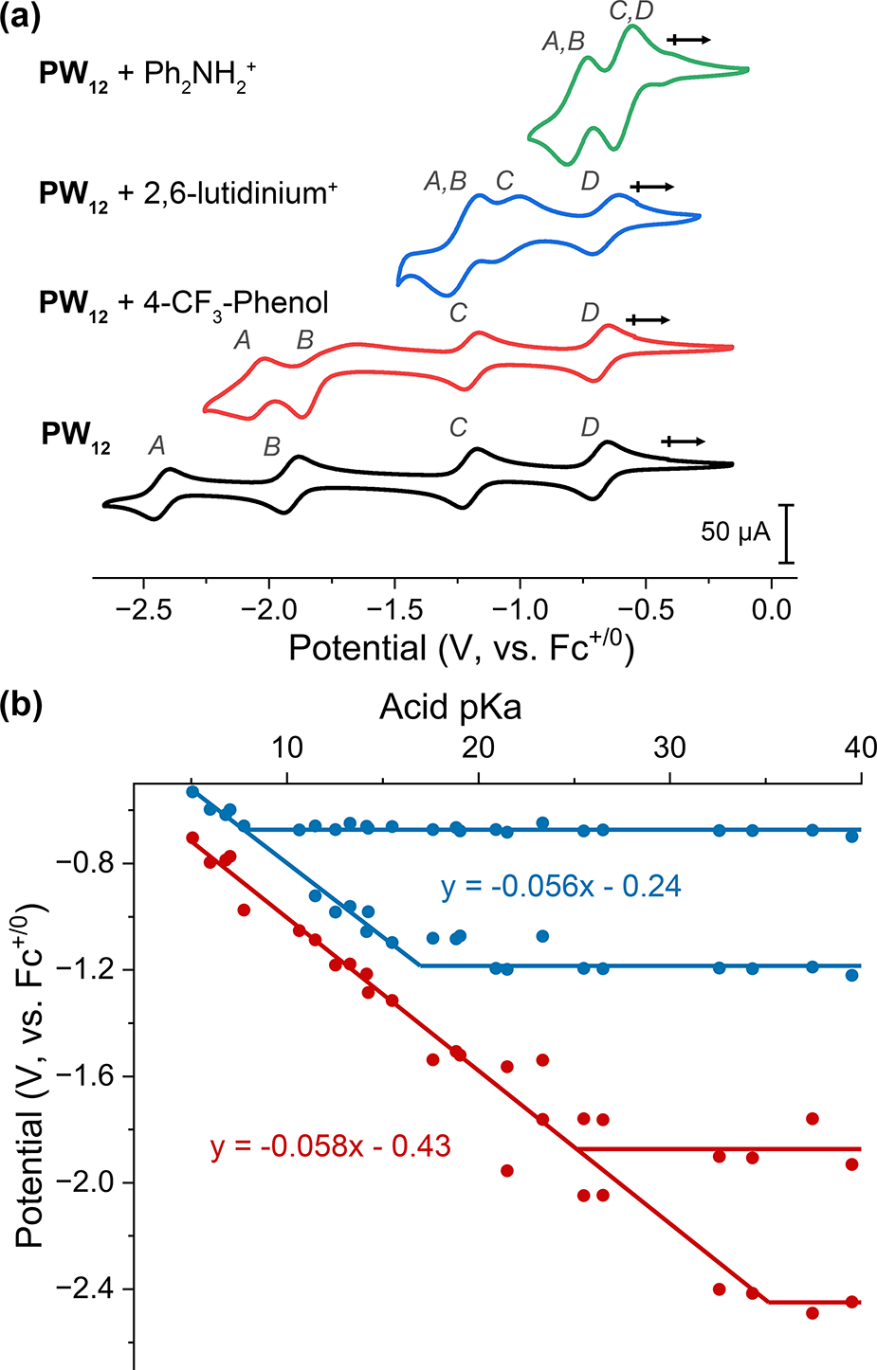
(a) CVs of 1 mM **PW**_**12**_ (black) and in the presence of 4 mM 4-trifluoromethylphenol (red, p*K*_a MeCN_ = 25.5), 2,6-lutidinium tetrafluoroborate (blue, p*K*_a MeCN_ = 14.16), and diphenylammonium tetrafluoroborate (green, p*K*_a MeCN_ = 5.98) in acetonitrile with 100 mM ^*n*^Bu_4_NPF_6_ at a scan rate of 200 mV/s. (b) Potential–p*K*_a_ diagram for **PW**_**12**_. All of the potentials are calibrated by using Fc^+/0^ as the internal standards. Each data point represents a CV collected in the presence of one acid; all other CVs are listed in Figure S1.

With the reduction potentials and p*K*_a_ values of reduced clusters in hand, the average BDFE(O–H) values for the observed multielectron/multiproton redox events can be calculated using the Bordwell equation (eq 1).
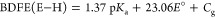
1where p*K*_a_ and *E*° are experimentally determined and *C*_g_ is a constant associated with the reduction of protons in acetonitrile. BDFE(O–H)_avg_ values of 48.1 and 43.7 kcal mol^–1^ are determined for the reduced and protonated forms of **PW**_**12**_ (see eq 2 and 3). These values are substantially smaller than those measured previously by our group for polyoxovanadate clusters.^31−36^ This trend is also broadly consistent with differences between the onset potentials for incipient H-insertion in bulk vanadium and tungsten oxides.^37−40^
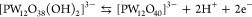
2
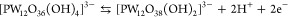
3

Based on the experimentally determined potential–p*K*_a_ diagram of **PW**_**12**_, we hypothesized that isolation of a mono- or bis-hydroxide substituted form of the POT cluster, [^*n*^Bu_4_N]_3_[PW_12_O_40–*x*_(OH)_*x*_] (*x* = 1, 2), would be possible upon addition of a chemical reductant (*E*_1/2_ < −0.8 V vs Fc^+/0^) to the Keggin ion in the presence of an appropriate acid (p*K*_a_ < 10). Instead, addition of a strong organic acid (e.g., H_2_NPh_2_^+^, p*K*_a MeCN_ = 5.98) to singly or doubly reduced forms of the cluster results in spontaneous formation of H_2_ (δ = 4.57 ppm) and reoxidation of the tungsten oxide assembly over the course of 3 h (Figure 3a, Figures S8 and S9). We note that the estimated BDFE(O–H)_avg_ of 48.1 kcal mol^–1^ is similar to that of H_2_ (BDFE(H–H) = 51.2 kcal mol^–1^ in acetonitrile).^22,41,42^ This suggests a thermodynamic equilibrium for H_2_ production from the surface of the reduced POT cluster in the presence of protons and further suggests that the activation barrier for uni- or bimolecular H–H bond formation is not prohibitively high.

**Figure 3:**
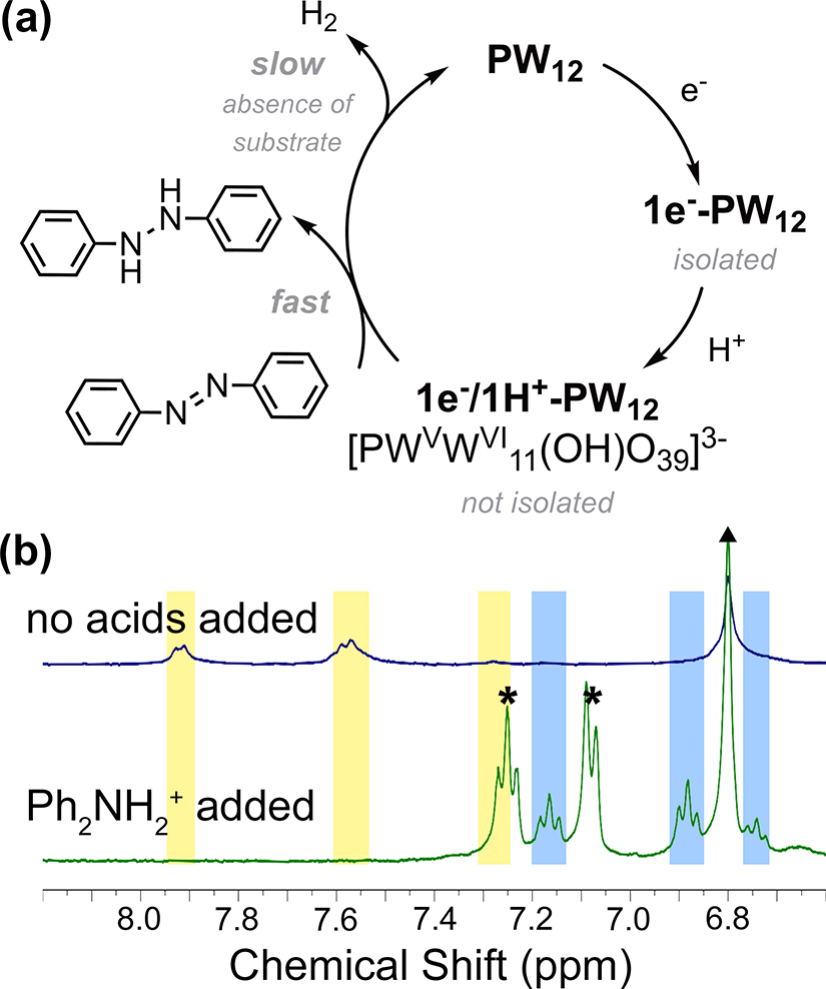
(a) Reaction scheme from **PW**_**12**_ to protonated, reduced cluster through **1e**^**–**^**-PW**_**12**_, semihydrogenation of azobenzene to hydrazobenzene, and spontaneous H_2_ formation through PCET processes. (b) ^1^H NMR spectra of the mixture of azobenzene and **1e**^**–**^**-PW**_**12**_ before and after the addition of the acid Ph_2_NH_2_BF_4_. The yellow-shaded region is the chemical shift for azobenzene, and the blue-shaded region is for hydrazobenzene. The triangles and asterisks indicate the internal standard mesitylene, and diphenylamine formed from Ph_2_NH_2_BF_4_.

Given the observed instability of the 1e^–^/1H^+^ reduced form of the POT cluster, we hypothesized that *in situ* proton uptake at the reduced **PW**_**12**_ cluster would serve as a source of H atoms for hydrogenation reactions. To isolate the reduced form of **PW**_**12**_, one equivalent of tetrabutylammonium borohydride (^*n*^Bu_4_NBH_4_) was added to a solution of the cluster in acetonitrile. An immediate color change from colorless to blue was observed. The singly reduced POT, [^*n*^Bu_4_N]_4_[PW^V^W^VI^_11_O_40_] (**1e**^**–**^**-PW**_**12**_) can be isolated from the reaction mixture via recrystallization (Figure 3a, Figure S4, see the SI for more information). In the electronic absorption spectrum **1e**^**–**^**-PW**_**12**_, a band centered at 765 nm (ε = 1855 M^–1^ cm^–1^) and a shoulder located at 494 nm (ε = 914 M^–1^ cm^–1^) are observed, which are both consistent with intervalence charge transfer (IVCT) bands.^43^^31^P NMR spectroscopy serves as an additional characterization method to distinguish the fully oxidized and one-electron reduced species (Figure S3).

With the reduced cluster in hand, we next targeted the hydrogenation of azobenzene to assess the feasibility of H atom transfer reactions using **PW**_**12**_ as the mediator (Figure 3a). Azobenzene can be reduced by 2e^–^/2H^+^ to form hydrazobenzene or by 4e^–^/4H^+^ to generate two equiv of aniline via N=N bond cleavage. Previous approaches to this hydrogenation, including photocatalysis and transition metal catalysis, realized semihydrogenation of azobenzene in moderate yields over extended time scales.^44−47^ But it is still challenging to achieve high conversion and selectivity for hydrazobenzene without sacrificing reaction rate.

Experimental conditions for the hydrogenation reactions are as follows: 1 equiv of azobenzene and 2 equiv of organic acid were combined with two equiv of prereduced **1e**^**–**^**-PW**_**12**_ in CD_3_CN, with mesitylene as the internal standard. First, azobenzene and **1e**^**–**^**-PW**_**12**_ are mixed (blue trace in Figure 3b). Upon the addition of a strong organic acid, H_2_NPh_2_^+^ (p*K*_a MeCN_ = 5.98), the mixture turns pale yellow within seconds, consistent with regeneration of the parent **PW**_**12**_. The ^1^H NMR spectrum, as shown as a green trace in Figure 3b, reveals the complete conversion of azobenzene and deprotonation of the organic acid. Although mixing **1e**^**–**^**-PW**_**12**_ and strong organic acid can generate dihydrogen gas, the larger difference in BDFE between hydrazobenzene (BDFE(N–H) = 60.9 kcal mol^–1^) and **PW**_**12**_ drives the more thermodynamically favored hydrogenation reaction over hydrogen evolution.^21,22,42^

Further kinetic experiments were conducted to probe the selectivity for azobenzene hydrogenation over hydrogen evolution. The difference in electronic absorption profiles of **1e**^**–**^**-PW**_**12**_ and **PW**_**12**_ enables monitoring of the reaction progression via the loss of the W(V) → W(VI) IVCT band of **1e**^**–**^**-PW**_**12**_ (λ = 765 nm). Azobenzene semihydrogenation is observed to proceed >1000 times faster than H_2_ formation even when the former is carried out at 60 °C lower temperature and 4-fold lower concentration of reagents (Figure S10). Additional kinetic measurements varying the initial concentration of reactants in azobenzene hydrogenation (see Kinetics section in the SI, Figures S11–S30) indicate that the hydrogenation reaction proceeds only in the presence of reduced cluster and acid, and the rate of azobenzene hydrogenation is dictated by the concentration of whichever of these two species is the limiting reagent.

To further rule out the possibility of stepwise reactivity involving sequential proton/electron transfer steps, NMR studies were conducted to probe for reactions between H_2_NPh_2_^+^ and azobenzene and between **1e**^**–**^**-PW**_**12**_ and azobenzene. No changes in the NMR spectra were observed in either case. This is broadly consistent with the thermodynamic properties of each species: electron transfer from **1e**^**–**^**-PW**_**12**_ to azobenzene is significantly endergonic, (Δ*G*_ET_ = +24.9 kcal mol^–1^, Figure S34) and Ph_2_NH_2_^+^ is not a strong enough acid to protonate azobenzene (the p*K*_a_ of protonated azobenzene is −2.95 in water).^48^

These observations lead us to conclude that the dominant pathway entails rapid formation of a reactive intermediate comprising the reduced and protonated POT bearing labile O–H bonds, followed by rate-determining H atom transfer to azobenzene. In the absence of azobenzene, formation of the same POT intermediate results in H–H bond formation and H_2_ evolution, albeit at a much slower rate. As to the identity of the POT intermediate, the potential vs p*K*_a_ relationships outlined in Figure 2 shows the singly reduced POT is unstable toward disproportionation (to form the doubly reduced/protonated cluster and **PW**_**12**_) in the presence H_2_NPh_2_^+^. Hence, the reactive POT intermediate may be the 2e^–^/2H^+^ transfer disproportionation product or a metastable 1e^–^/1H^+^ transfer product. Notably, analytical methods used in this study do not enable straightforward differentiation between these species.

The observed selectivity for azobenzene hydrogenation over hydrogen evolution is broadly consistent with markedly different observed rates of the respective reactions, which can in turn be attributed to the much larger driving force for substrate hydrogenation compared to hydrogen evolution—that is, hydrogenated azobenzene is the thermodynamic product. However, full hydrogenation of azobenzene to aniline is also more thermodynamically favorable than semihydrogenation. Thus, thermodynamics alone cannot explain the selective production of hydrazobenzene. An alternative rationale is that the full hydrogenation reaction proceeds sequentially through hydrazobenzene, so conditions involving the addition of only two equiv of hydrogen (via **1e**^**–**^**-PW**_**12**_ and Ph_2_NH_2_^+^) self-limit at the semihydrogenation product. However, we observe no aniline formation even in the presence of 4 equiv of **1e**^**–**^**-PW**_**12**_ and Ph_2_NH_2_^+^ (Figure S33). Instead, after converting azobenzene into hydrazobenzene, the remaining **1e**^**–**^**-PW**_**12**_ and acid react to generate dihydrogen rather than to further hydrogenate the hydrazobenzene. This leaves only kinetic control—i.e., markedly higher activation energies for hydrazobenzene hydrogenation compared to azobenzene hydrogenation—as the most plausible explanation for the observed selectivity.

To summarize, we have leveraged PCET reactivity of **PW**_**12**_ for the stoichiometric semihydrogenation of azobenzene by independently controlling the availability of electrons (via chemical formation of the singly reduced cluster **1e**^**–**^**-PW**_**12**_) and protons (via control over the concentration and p*K*_a_ of the organic acid). This type of control was enabled through a complete mapping of the potential–p*K*_a_ relationships (Figure 2b) associated with proton/electron transfer to the cluster. This approach further allowed us to identify reaction conditions under which the cluster could be used to deliver quantitative semihydrogenation of the model H-acceptor azobenzene, where the exclusion of H_2_ and aniline as byproducts can be attributed to thermodynamic and kinetic effects, respectively.

## Supporting Information Available

Electrochemical data, characterization **1e**^–^**-PW**_**12**_, reactivity of **1e**^–^**-PW**_**12**_, and kinetic data(PDF)
