# Supplementary material for: Selective Hydrogenation of Azobenzene to Hydrazobenzene via Proton-Coupled Electron Transfer from a Polyoxotungstate Cluster
Source: JACS Au. 2024 Mar 21;4(4):1310–4. doi: 10.1021/jacsau.4c00127 (PMC11041919; doi:10.1021/jacsau.4c00127)
Supplement: Supplementary file 1 — au4c00127_si_001.pdf [file au4c00127_si_001.pdf]

Electronic Supporting Information File for

Selective Hydrogenation of Azobenzene to Hydrazobenzene via Proton-Coupled Electron Transfer from a Polyoxotungstate Cluster

Zhou Lu[1], Shannon E. Cooney[1], James R. McKone[2]*, Ellen M. Matson[1]*

[1]Department of Chemistry, University of Rochester, Rochester 14627, New York, United States

*Corresponding Author E-mail: matson@chem.rochester.edu

[2]Department of Chemical and Petroleum Engineering, University of Pittsburgh, Pittsburgh, Pennsylvania 15260, United States.

*Corresponding Author E-mail: jmckone@pitt.edu

Table of Contents

General Considerations and Physical Measurements. 4
General procedure for performing pseudo-first-order reaction mechanism/kinetics. 4
Synthesis of $[^nBu_4N]_4[PW^VW^{VI}_{11}O_{40}]$ (**$1e^-$-PW_{12}**). 6
Figure S1. Cyclic voltammograms of 1 mM **PW_{12}** obtained in acetonitrile in the presence of 4 mM various organic acids with the scan rate of 200 mV/s, using 0.1 M $[^nBu_4N]PF_6$ as the supporting electrolyte. Ferrocene is used for each measurement as the internal standard. The corresponding acids are listed in **Table S1**. 7
Table S1. pK_a values of various organic acids in acetonitrile. 8
Figure S2. ^{1}H-NMR spectrum of **$1e^-$-PW_{12}** in CD_3CN at room temperature, 21 °C. 9
Figure S3. ^{31}P-NMR spectra of **PW_{12}** and **$1e^-$-PW_{12}** in CD_3CN at room temperature, 21 °C. 9
Figure S4. Electronic absorption spectra of **PW_{12}** and **$1e^-$-PW_{12}** in MeCN. Inset figure is the zoom-in wavelength region from 400 to 1000 nm. 9
Figure S5. FT-IR spectrum of **$1e^-$-PW_{12}** in solid state, at room temperature, 21 °C. 10
Figure S6. Cyclic voltammogram of 1 mM **$1e^-$-PW_{12}** in acetonitrile with 100 mM $[^nBu_4N]PF_6$ supporting electrolyte. The scan rate is 200 mV/s. The potential is calibrated by using $Fc^{+/0}$ as the internal standard. 10
Figure S7. (a) Cyclic voltammograms of 1 mM **$1e^-$-PW_{12}** with 0.1 M $[^nBu_4N]PF_6$ at scan rates from 10 – 1000 mV/s in acetonitrile. (b) Randles–Sevcik analysis of multiple redox couples **$1e^-$-PW_{12}** with 0.1 M $[^nBu_4N]PF_6$ in acetonitrile. 11
Figure S8. ^{1}H-NMR of the evolution of dihydrogen (H_2) from the mixture of **$1e^-$-PW_{12}** and strong acid diphenylammonium tetrafluoroborate ($Ph_2NH_2^+$, pK_a(MeCN) = 5.98) in CD_3CN at room temperature, 21 °C. 11
Figure S9. The relative integration area of H_2 evolution (4.52 - 4.60 ppm) from the mixture of **$1e^-$-PW_{12}** and one strong acid diphenylammonium tetrafluoroborate ($Ph_2NH_2^+$, pK_a(MeCN) = 5.98) over the 4-hour time period. 12
Figure S10. Normalized absorbance (λ= 765 nm, characteristic absorption of reduced **PW_{12}** cluster) changes over the time. 12
Figure S11. (Left) Plot of observed pseudo-1st-order rate constant (k_{obs}) as a function of [azobenzene] in MeCN at -35 °C. (Right) Plot of natural log of observed pseudo-1st-order rate constant (k_{obs}) as a function of natural log of [azobenzene] in MeCN at -35 °C. 13
Figure S12. Plot of absorbance at 765 nm over time for reactions between azobenzene (0.3 mM), $Ph_2NH_2^+$ (5 mM), and **$1e^-$-PW_{12}** (0.05 mM) under pseudo-1st-order conditions recorded in MeCN at -35 °C with (grey) raw data and (red) fit curve, along with the fit-derived k_{obs} and R^2 parameters. 13
Figure S13. Plot of absorbance at 765 nm over time for reactions between azobenzene (0.5 mM), $Ph_2NH_2^+$ (5 mM), and **$1e^-$-PW_{12}** (0.05 mM) under pseudo-1st-order conditions recorded in MeCN at -35 °C with (grey) raw data and (red) fit curve, along with the fit-derived k_{obs} and R^2 parameters. 13
Figure S14. Plot of absorbance at 765 nm over time for reactions between azobenzene (0.6 mM), $Ph_2NH_2^+$ (5 mM), and **$1e^-$-PW_{12}** (0.05 mM) under pseudo-1st-order conditions recorded in MeCN at -35 °C with (grey) raw data and (red) fit curve, along with the fit-derived k_{obs} and R^2 parameters. 14
Figure S15. Plot of absorbance at 765 nm over time for reactions between azobenzene (0.8 mM), $Ph_2NH_2^+$ (5 mM), and **$1e^-$-PW_{12}** (0.05 mM) under pseudo-1st-order conditions recorded in MeCN at -35 °C with (grey) raw data and (red) fit curve, along with the fit-derived k_{obs} and R^2 parameters. 14
Figure S16. Plot of observed pseudo-1st-order rate constant (k_{obs}) as a function of [**$1e^-$-PW_{12}**] in MeCN at -35 °C. 14
Figure S17. Plot of absorbance at 765 nm over time for reactions between **$1e^-$-PW_{12}** (0.2 mM), azobenzene (5 mM), and $Ph_2NH_2^+$ (0.05 mM) under pseudo-1st-order conditions recorded in MeCN at -35 °C with (grey) raw data and (red) fit curve, along with the fit-derived k_{obs} and R^2 parameters. 15
Figure S18. Plot of absorbance at 765 nm over time for reactions between **$1e^-$-PW_{12}** (0.3 mM), azobenzene (5 mM), and $Ph_2NH_2^+$ (0.05 mM) under pseudo-1st-order conditions recorded in MeCN at -35 °C with (grey) raw data and (red) fit curve, along with the fit-derived k_{obs} and R^2 parameters. 15
Figure S19. Plot of absorbance at 765 nm over time for reactions between **$1e^-$-PW_{12}** (0.4 mM), azobenzene (5 mM), and $Ph_2NH_2^+$ (0.05 mM) under pseudo-1st-order conditions recorded in MeCN at -35 °C with (grey) raw data and (red) fit curve, along with the fit-derived k_{obs} and R^2 parameters. 15

Figure S20. Plot of absorbance at 765 nm over time for reactions between **$1e^-$-PW_{12}** (0.5 mM), azobenzene (5 mM), and $Ph_2NH_2^+$ (0.05 mM) under pseudo-1st-order conditions recorded in MeCN at -35 °C with (grey) raw data and (red) fit curve, along with the fit-derived k_{obs} and R^2 parameters. 16
Figure S21. Plot of observed pseudo-1st-order rate constant (k_{obs}) as a function of [$Ph_2NH_2^+$] in MeCN at -35 °C. 16
Figure S22. Plot of absorbance at 765 nm over time for reactions between $Ph_2NH_2^+$ (0.5 mM), azobenzene (5 mM), and **$1e^-$-PW_{12}** (0.05 mM) under pseudo-1st-order conditions recorded in MeCN at -35 °C with (grey) raw data and (red) fit curve, along with the fit-derived k_{obs} and R^2 parameters. 16
Figure S23. Plot of absorbance at 765 nm over time for reactions between $Ph_2NH_2^+$ (0.6 mM), azobenzene (5 mM), and **$1e^-$-PW_{12}** (0.05 mM) under pseudo-1st-order conditions recorded in MeCN at -35 °C with (grey) raw data and (red) fit curve, along with the fit-derived k_{obs} and R^2 parameters. 17
Figure S24. Plot of absorbance at 765 nm over time for reactions between $Ph_2NH_2^+$ (0.8 mM), azobenzene (5 mM), and **$1e^-$-PW_{12}** (0.05 mM) under pseudo-1st-order conditions recorded in MeCN at -35 °C with (grey) raw data and (red) fit curve, along with the fit-derived k_{obs} and R^2 parameters. 17
Figure S25. Plot of absorbance at 765 nm over time for reactions between $Ph_2NH_2^+$ (1.2 mM), azobenzene (5 mM), and **$1e^-$-PW_{12}** (0.05 mM) under pseudo-1st-order conditions recorded in MeCN at -35 °C with (grey) raw data and (red) fit curve, along with the fit-derived k_{obs} and R^2 parameters. 17
Figure S26. (Left) Plot of observed pseudo-1st-order rate constant (k_{obs}) as a function of [$Ph_2NH_2^+$] or [**$1e^-$-PW_{12}**] (the concentration of in situ generated protonated, reduced $[PW_{12}O_{39}(OH)]^{3-}$) in MeCN at -35 °C. (Right) Plot of natural log of observed pseudo-1st-order rate constant (k_{obs}) as a function of natural log of [$Ph_2NH_2^+$] or [**$1e^-$-PW_{12}**] (the concentration of in situ generated protonated, reduced $[PW_{12}O_{39}(OH)]^{3-}$) in MeCN at -35 °C. 18
Figure S27. Plot of absorbance at 765 nm over time for reactions between azobenzene (5 mM), $Ph_2NH_2^+$ (0.05 mM), and **$1e^-$-PW_{12}** (0.05 mM) under pseudo-1st-order conditions recorded in MeCN at -35 °C with (grey) raw data and (red) fit curve, along with the fit-derived k_{obs} and R^2 parameters. 18
Figure S28. Plot of absorbance at 765 nm over time for reactions between azobenzene (5 mM), $Ph_2NH_2^+$ (0.075 mM), and **$1e^-$-PW_{12}** (0.075 mM) under pseudo-1st-order conditions recorded in MeCN at -35 °C with (grey) raw data and (red) fit curve, along with the fit-derived k_{obs} and R^2 parameters. 18
Figure S29. Plot of absorbance at 765 nm over time for reactions between azobenzene (5 mM), $Ph_2NH_2^+$ (0.1 mM), and **$1e^-$-PW_{12}** (0.1 mM) under pseudo-1st-order conditions recorded in MeCN at -35 °C with (grey) raw data and (red) fit curve, along with the fit-derived k_{obs} and R^2 parameters. 19
Figure S30. Plot of absorbance at 765 nm over time for reactions between azobenzene (5 mM), $Ph_2NH_2^+$ (0.125 mM), and **$1e^-$-PW_{12}** (0.125 mM) under pseudo-1st-order conditions recorded in MeCN at -35 °C with (grey) raw data and (red) fit curve, along with the fit-derived k_{obs} and R^2 parameters. 19
Figure S31. ^{31}P-NMR spectra of the mixture of **$1e^-$-PW_{12}**, azobenzene, and (top) weak acid benzoic acid (pKa(MeCN) = 21.5) or (bottom) strong acid diphenylammonium tetrafluoroborate ($Ph_2NH_2^+$, pKa(MeCN) = 5.98) at room temperature, 21 °C. 20
Figure S32. ^{1}H NMR spectra of (top) the mixture of 2 equivalents of $Ph_2NH_2^+$ and 1 equivalent of azobenzene, (middle) the mixture of 2 equivalents of **$1e^-$-PW_{12}** and 1 equivalent of azobenzene, and (bottom) azobenzene only in CD_3CN at room temperature, 21 °C. 20
Figure S33. ^{1}H NMR spectra of (top) the mixture of 4 equivalents of **$1e^-$-PW_{12}** and $Ph_2NH_2^+$, 1 equivalent of azobenzene and (bottom) aniline only in CD_3CN at room temperature, 21 °C. 20
Figure S34. Cyclic voltammograms of 1 mM azobenzene obtained in acetonitrile with the scan rate of 100 mV/s, using 100 mM [$^nBu_4N$]PF_6 as the supporting electrolyte. 21
References 22

General Considerations and Physical Measurements.

All the experiments were carried out in a UniLab MBraun inert atmosphere glove box under a dinitrogen gas atmosphere. All glassware was oven-dried and cooled in an evacuated antechamber prior to use. Solvents were dried and deoxygenated on a glass contour system (Pure Process Technology, LLC) and stored over 3 Å molecular sieves that are activated prior to use. The deuterated acetonitrile was freeze-pump-thawed for at least three cycles and stored over 3 Å molecular sieves in an oven-dried Schlenk flask before moving into the dry box. All chemicals were used as received. The $[^{n}Bu_4N]_3[PW^{VI}{}_{12}O_{40}]$ (**PW_{12}**) cluster was synthesized according to the literature.[1]

Electronic absorption spectroscopy was recorded in anhydrous acetonitrile in 1-cm-path quartz cuvette with an Agilent Cary 60 UV-Vis Spectrophotometer at room temperature. Elemental analysis was performed on a PerkinElmer 2400 Series II CHNS/O Elemental Analyzer. NMR studies were carried out on a Brüker 400 MHz spectrometer. The concentrations of each species used in this work were calibrated by using the 10 mM mesitylene as the internal standard. ^{31}P NMR was externally calibrated by adopting the chemical shift of H_3PO_4 in deuterated acetonitrile as 0 ppm. Infrared spectroscopy was conducted with a Perkin Elmer Spectrum 3 FT-IR Spectrometer.

All electrochemistry measurements were performed by using a BioLogic SP-150 Potentiostat and acquired with the EC-Lab software (V11.42). Glassy carbon disc (3 mm, CH Instruments, USA) and a platinum wire was used as working and counter electrode, respectively. A nonaqueous Ag/Ag^{+} reference electrode with 1 mM $AgNO_3$ and 100 mM $[^{n}Bu_4N]PF_6$ in acetonitrile (BASi, USA) was used as the reference electrode. All cyclic voltammetry (CV) measurements were carried out at room temperature in a nitrogen-filled glove box and calibrated by $Fc^{+/0}$ couple. The scan rate is 200 mV/s unless otherwise specified. The potential-pK_a diagram of **PW_{12}** was established by measuring the CV of the mixture of 1 mM **PW_{12}** and 4 mM organic acid in the corresponding scan windows; all the potentials were finally calibrated by adding $Fc^{+/0}$ into the mixture as the internal standard and then collecting an additional CV. The pKa equations can be expressed as follows:

$[PW_{12}O_{39}(OH)]^{3-} \leftrightarrows [PW_{12}O_{40}]^{3-} + H^{+} + e^{-}$ (Eqn. S1)

$[PW_{12}O_{38}(OH)_2]^{3-} \leftrightarrows [PW_{12}O_{39}(OH)]^{3-} + H^{+} + e^{-}$ (Eqn. S2)

$[PW_{12}O_{37}(OH)_3]^{3-} \leftrightarrows [PW_{12}O_{38}(OH)_2]^{3-} + H^{+} + e^{-}$ (Eqn. S3)

$[PW_{12}O_{36}(OH)_4]^{3-} \leftrightarrows [PW_{12}O_{37}(OH)_3]^{3-} + H^{+} + e^{-}$ (Eqn. S4)

The BDFE values are calculated by adopting Eqn. 1. The pKa values are 34.8 ± 0.6 (pK_a1, Eqn S1), 25.9 ± 0.4 (pK_a2, Eqn S2), 17.4 ± 0.4 (pK_a3, Eqn S3), and 8.3 ± 0.2 (pK_a4, Eqn S4); the respective corresponding E1/2 values are -2.45, -1.93, -1.22, and -0.70 V vs $Fc^{+/0}$. The constant Cg in acetonitrile is 52.6 kcal mol^{-1}. The calculated BDFE values are 43.8, 43.6, 48.3, and 47.8 kcal mol^{-1}. As the redox events A and B, C and D would combine into a multi-proton/multi-electron event (Figure 2a, Figure S1), the BDFE values calculated for events A and B, C and D are averaged to obtain 43.7 and 48.1 kcal mol^{-1}.

General procedure for performing pseudo-first-order reaction mechanism/kinetics.

Pseudo-First order reaction conditions were used in order to establish the rate expression for the reaction between the reaction between **$1e^{-}$-PW_{12}**, $Ph_2NH_2^{+}$, and azobenzene. To determine the order of each reactant with respect to the rate expression, k_{obs} was determined using UV-vis spectroscopy, where the loss of **$1e^{-}$-PW_{12}** can be measured over time. Upon completion of the reaction, the plot of absorbance over time was fit to the following equation by least squares fitting:

$$A_t = A_{inf} + (A_0 - A_{inf})e^{-k_{obs} \times t}$$

where A_t is the absorbance at a given time, t, in seconds, A_{inf} is the absorbance at the end of the reaction (t = infinite), A_0 is the absorbance after reductant injection, and k_{obs} is the observed first order rate constant (s^{-1}). Error was determined by calculating the standard deviation of k_{obs} between triplicate trials.

To find the order with respect to azobenzene, a sample of acetonitrile (MeCN) containing 0.05 mM of **1e$^-$-PW$_{12}$** and 5 mM of azobenzene was prepared in a long-necked air-free quartz cuvette with a stir bar sealed with a rubber septum. The sample was then removed from the glovebox and placed in the instrument to cool to –35 °C. A syringe containing $Ph_2NH_2^+$ was removed from the glovebox (40 mM stock solution). Once the sample reached desired temperature, collection of data began at 765 nm and the stock solution of $Ph_2NH_2^+$ was injected into the cuvette (final volume of 4 mL) to begin the reaction. The absorbance at 765 nm was collected every 0.0125 s until the reaction was complete. Each trial was repeated in triplicate. The reaction was then repeated using a different concentration of azobenzene (0.3 – 0.8 mM). A plot of k_{obs} vs [azobenzene] shows the linear relationship and another plot of natural log of k_{obs} vs natural log of [azobenzene] shows the slope of 0.95, close to 1 and confirming the reaction is first-order with respect to azobenzene (Figure S11).

To find the order with respect to **1e$^-$-PW$_{12}$**, a sample of acetonitrile (MeCN) containing various concentrations (0.2 – 0.5 mM) of **1e$^-$-PW$_{12}$** and 5 mM of azobenzene was prepared in a long-necked air-free quartz cuvette with a stir bar sealed with a rubber septum. The sample was then removed from the glovebox and placed in the instrument to cool to –35 °C with stirring. A syringe containing 0.1 mL $Ph_2NH_2^+$ solution was removed from the glovebox (2 mM stock solution). Once the sample reached desired temperature, collection of data began at 765 nm and the stock solution of $Ph_2NH_2^+$ was injected into the cuvette (final volume of 4.0 mL) to begin the reaction. The absorbance at 765 nm was collected every 0.0125 s until the reaction was complete. Each trial was repeated in triplicate. A plot of k_{obs} vs [**1e$^-$-PW$_{12}$**] reveals no change in rate as a function of concentration of the cluster, indicating the reaction is zeroth order with respect to **1e$^-$-PW$_{12}$** (Figure S16).

To find the order with respect to $Ph_2NH_2^+$, a sample of acetonitrile (MeCN) containing 0.05 mM of **1e$^-$-PW$_{12}$** and 5 mM of azobenzene was prepared in a long-necked air-free quartz cuvette with a stir bar sealed with a rubber septum. The sample was then removed from the glovebox and placed in the instrument to cool to –35 °C. A syringe containing $Ph_2NH_2^+$was removed from the glovebox (25 mM stock solution). Once the sample reached desired temperature, collection of data began at 765 nm and the stock solution of $Ph_2NH_2^+$ was injected into the cuvette (final volume of 4.0 mL) to begin the reaction. The absorbance at 765 nm was collected every 0.0125 s until the reaction was complete. Each trial was repeated in triplicate. The reaction was then repeated using a different concentration of $Ph_2NH_2^+$ (0.5 – 1.2 mM). A plot of k_{obs} vs [$Ph_2NH_2^+$] reveals no change in rate as a function of concentration of the cluster, indicating the reaction is zeroth order with respect to $Ph_2NH_2^+$ (Figure S21).

The rate orders of the cluster **1e$^-$-PW$_{12}$** and acid ($Ph_2NH_2^+$) are determined as zero, indicating the limiting reagent of hydrogenation reaction of azobenzene is the *in situ* generated protonated, reduced $[PW_{12}O_{39}(OH)]^{3-}$ species. The overdose of **1e$^-$-PW$_{12}$** or $Ph_2NH_2^+$ is independent to the k_{obs}, suggesting the concerted proton-electron transfer to the substrate. In this manner, the concentrations of **1e$^-$-PW$_{12}$** or $Ph_2NH_2^+$ are changed synchronously to find the rate order with respect to *in situ* generated protonated, reduced $[PW_{12}O_{39}(OH)]^{3-}$. A sample of acetonitrile containing 0.05 – 0.125 mM of **1e$^-$-PW$_{12}$** and 5 mM of azobenzene was prepared in a long-necked air-free quartz cuvette with a stir bar sealed with a rubber septum. The sample was then removed from the glovebox and placed in the instrument to cool to –35 °C. A syringe containing $Ph_2NH_2^+$was removed from the glovebox (2 mM stock solution). Once the sample reached desired temperature, collection of data began at 765 nm and the stock solution of $Ph_2NH_2^+$ was injected into

the cuvette (final volume of 4.0 mL) to begin the reaction. The absorbance at 765 nm was collected every 0.0125 s until the reaction was complete. Each trial was repeated in triplicate. The reaction was then repeated using different concentrations of **1e$^-$-PW$_{12}$** and $Ph_2NH_2^+$ (0.05 – 0.125 mM). A plot of k_{obs} vs [**1e$^-$-PW$_{12}$**] and [$Ph_2NH_2^+$] reveals the linear relationship, and another plot of natural log of k_{obs} vs natural log of [**1e$^-$-PW$_{12}$**] and [$Ph_2NH_2^+$] shows the slope of 0.88, revealing the reaction is first-order with respect to $[PW_{12}O_{39}(OH)]^{3-}$ (Figure S26).

Synthesis of [^{n}Bu$_4$N]$_4$[PW^{V}W^{VI}$_{11}$O$_{40}$] (**1e$^-$-PW$_{12}$**).

A 20-mL scintillation vial was charged with [^{n}Bu$_4$N]$_3$[PW^{VI}$_{12}$O$_{40}$] (**PW$_{12}$**, 0.250 g, 0.069 mmol) and [^{n}Bu$_4$N]BH$_4$ (0.020 g, 0.077 mmol) dissolved in ~10 mL acetonitrile. The reaction mixture was stirred at 50 °C overnight to afford a deep cyan solution. The solution was then concentrated under reduced pressure (~15 %), followed by layering with diethyl ether (5×3 mL) to re-crystallize the product. The cyan powder was then filtered, washed with diethyl ether, and dried under vacuum. Yield = 0.226 g, 84.7% based on **PW$_{12}$**. ^{1}H NMR (400 MHz, CD_3CN), δ = 3.16 (t, 32H), 1.66 (quintet, 32H), 1.42 (sextet, 32H), 1.00 (t, 48H). ^{31}P NMR (400 MHz, CD_3CN), δ = -12.54 ppm. Anal. Calcd. for $PW_{12}O_{40}C_{64}H_{144}N_4$: C, 19.98%; H, 3.77%; N, 1.46%; found: C, 20.158%; H: 3.577%; N, 1.517%.

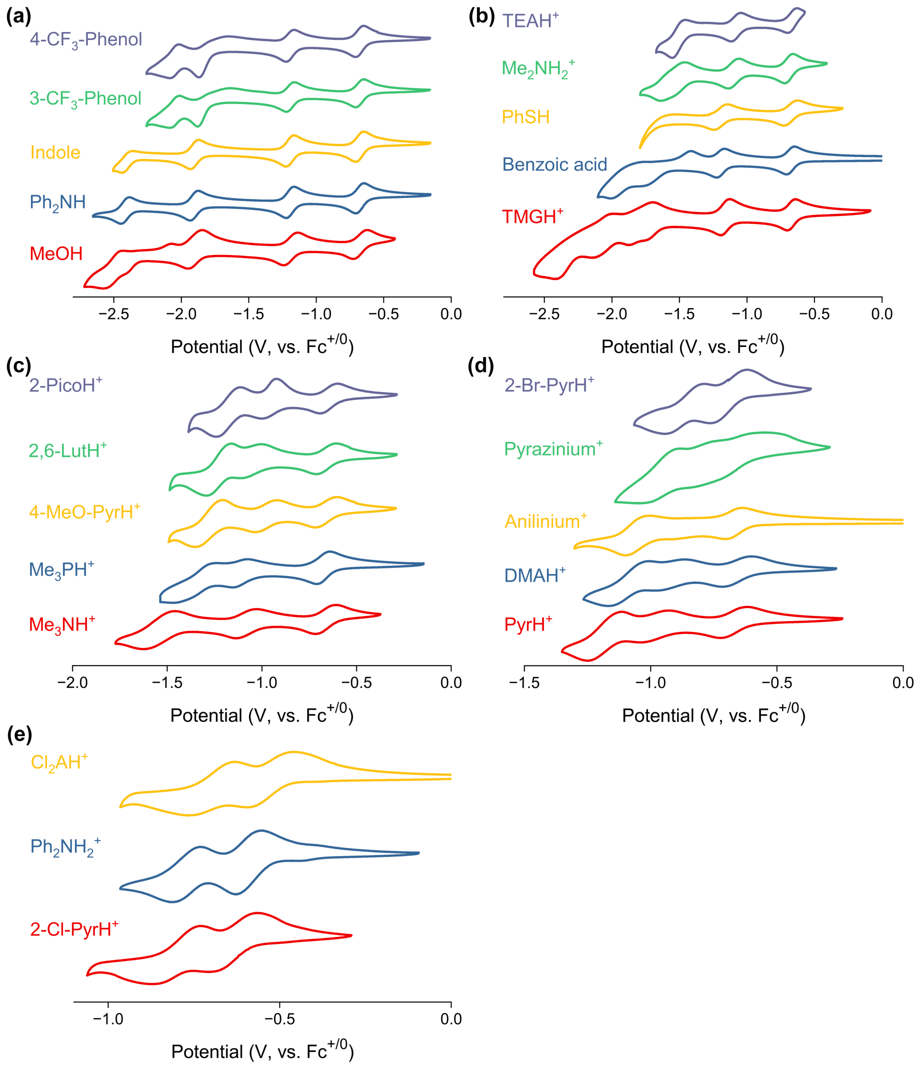

Figure S1. Cyclic voltammograms of 1 mM **PW_{12}** obtained in acetonitrile in the presence of 4 mM various organic acids with the scan rate of 200 mV/s, using 0.1 M [$^nBu_4N$]PF_6 as the supporting electrolyte. Ferrocene is used for each measurement as the internal standard. The corresponding acids are listed in **Table S1**.

Table S1. pK_a values of various organic acids in acetonitrile.

Acid	Abbreviation	pK_a(MeCN)	Ref.
Acetonitrile	MeCN	39.5	2,3
Methanol	MeOH	37.44	2,4
Diphenylamine	Ph_2NH	34.3	2,5
Indole		32.57	6
3-Trifluoromethyl-Phenol	3-CF3-phenol	26.5	6
4-Trifluoromethyl-Phenol	4-CF3-phenol	25.5	6
1,1,3,3-Tetramethylguanidinium tetrafluoroborate	$TMGH^+$	23.35	7
Benzoic acid		21.5	8
Thiophenol	PhSH	20.91	2,9
Dimethylammonium chloride	$Me_2NH_2^+$	19.03	7
Triethylammonium tetrafluoroborate	$TEAH^+$	18.83	7
Trimethylammonium chloride	Me_3NH^+	17.61	7
Trimethylphosphonium tetrafluoroborate	Me_3PH^+	15.48	7
4-Methoxyl-Pyridium tetrafluoroborate	4-MeO-$PyrH^+$	14.24	7
2,6-Lutidinium tetrafluoroborate	2,6-$LutH^+$	14.16	7
2-Picodinium tetrafluoroborate	2-$PicoH^+$	13.28	7
Pyridium tetrafluoroborate	$PyrH^+$	12.53	7
N,*N*-Dimethylanilinium tetrafluoroborate	$DMAH^+$	11.47	7
Anilinium tetrafluoroborate	$Anilinium^+$	10.64	7
Pyrazinium tetrafluoroborate	$Pyrazinium^+$	7.74	7
2-Bromo-Pyridium tetrafluoroborate	2-Br-$PyrH^+$	7.02	7
2-Chloro-Pyridium tetrafluoroborate	2-Cl-$PyrH^+$	6.79	7
Diphenylammonium tetrafluoroborate	$Ph_2NH_2^+$	5.98	7
2,6-Dichloro-Anilinium tetrafluoroborate	Cl_2AH^+	5.07	7

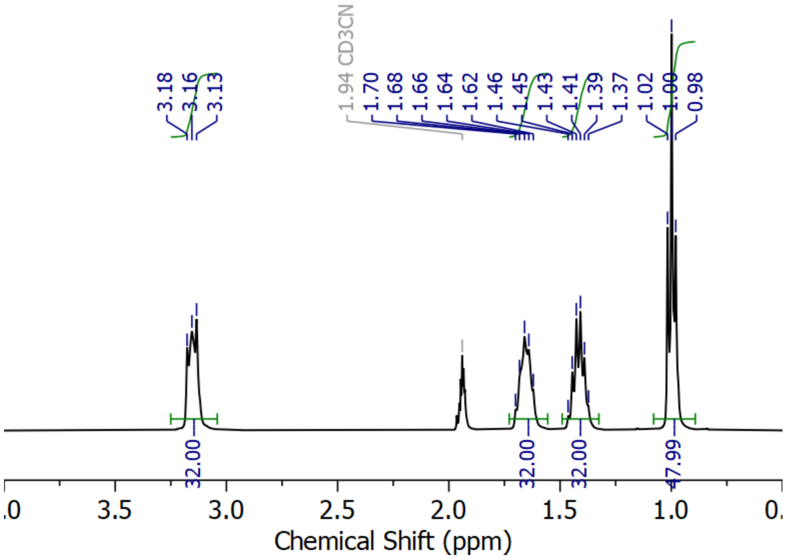

Figure S2. ^{1}H-NMR spectrum of **1e$^-$-PW$_{12}$** in CD_3CN at room temperature, 21 °C.

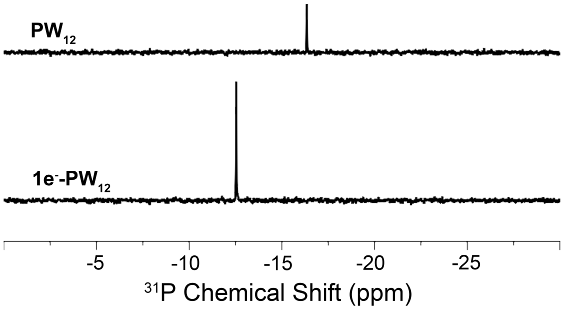

Figure S3. ^{31}P-NMR spectra of **PW$_{12}$** and **1e$^-$-PW$_{12}$** in CD_3CN at room temperature, 21 °C. The phosphoric acid in CD_3CN is used as the external standard by calibrating at 0 ppm.

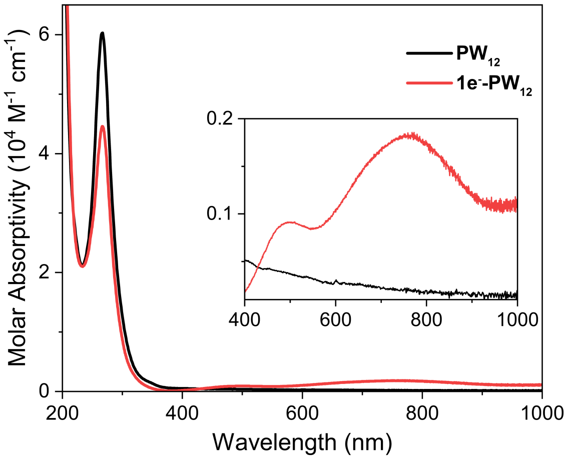

Figure S4. Electronic absorption spectra of **PW$_{12}$** and **1e$^-$-PW$_{12}$** in MeCN. Inset figure is the zoom-in wavelength region from 400 to 1000 nm.

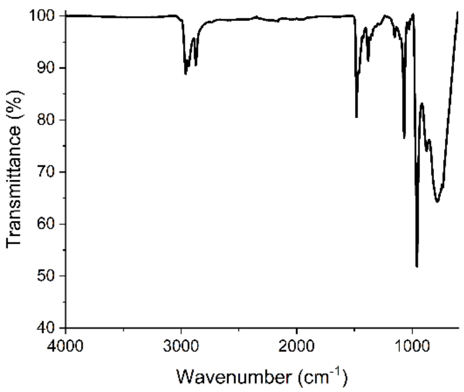

Figure S5. FT-IR spectrum of **1e^{-}-PW$_{12}$** in solid state, at room temperature, 21 °C.

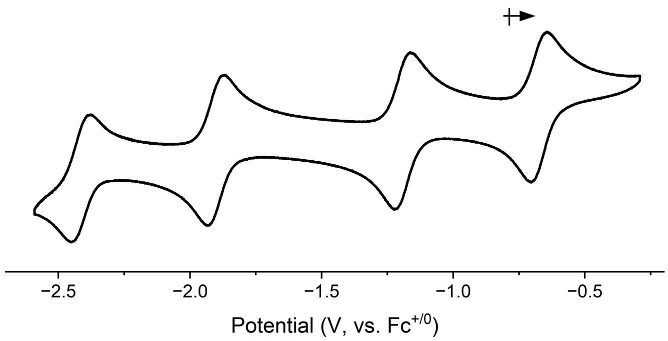

Figure S6. Cyclic voltammogram of 1 mM **1e^{-}-PW$_{12}$** in acetonitrile with 100 mM [^{n}Bu$_4$N]PF$_6$ supporting electrolyte. The scan rate is 200 mV/s. The potential is calibrated by using Fc$^{+/0}$ as the internal standard. Open circuit potential and scan direction are indicated by the black arrow.

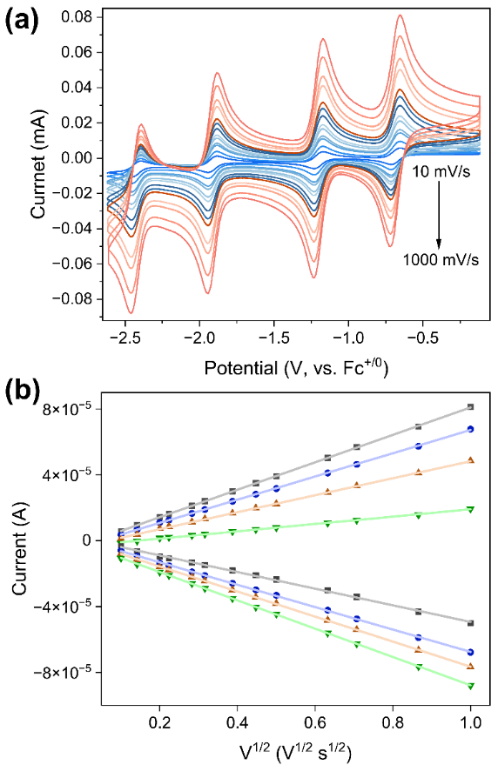

Figure S7. (a) Cyclic voltammograms of 1 mM **$1e^-$-PW$_{12}$** with 0.1 M [^{n}Bu$_4$N]PF$_6$ at scan rates from 10 – 1000 mV/s in acetonitrile. (b) Randles–Sevcik analysis of multiple redox couples **$1e^-$-PW$_{12}$** with 0.1 M [^{n}Bu$_4$N]PF$_6$ in acetonitrile.

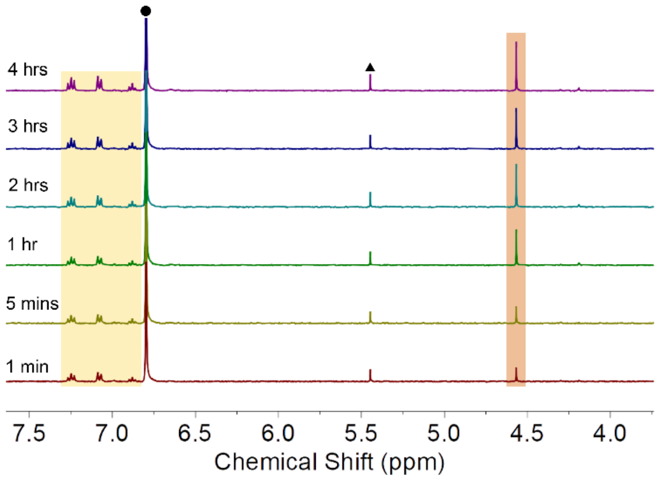

Figure S8. ^{1}H-NMR of the evolution of dihydrogen (H_2) from the mixture of **$1e^-$-PW$_{12}$** and strong acid diphenylammonium tetrafluoroborate ($Ph_2NH_2^+$, pK_a(MeCN) = 5.98) in CD_3CN at room temperature, 21 °C. The orange-shaded region is the chemical shift for H_2 gas, and the yellow-shaded region is the chemical shifts of generated conjugate base diphenylamine (Ph_2NH). The circle represents the internal standard mesitylene and the triangle is the trace impurity from the micropipette tips.

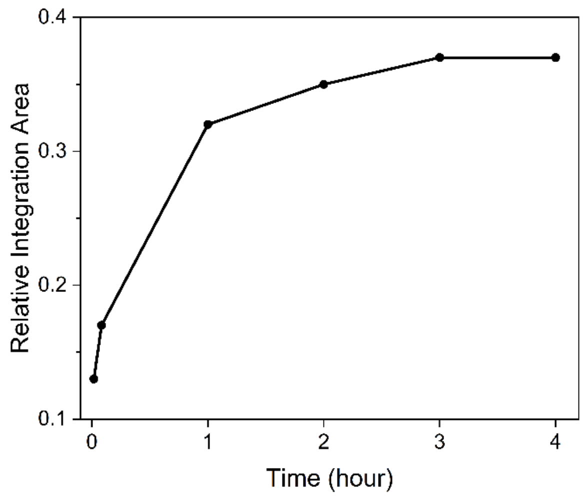

Figure S9. The relative integration area of H_2 evolution (4.52 - 4.60 ppm) from the mixture of **$1e^-$-PW_{12}** and one strong acid diphenylammonium tetrafluoroborate ($Ph_2NH_2^+$, pK_a(MeCN) = 5.98) over the 4-hour time period. The integration area is relative to the chemical shift (6.70 – 6.83 ppm) of internal standard mesitylene as 3.

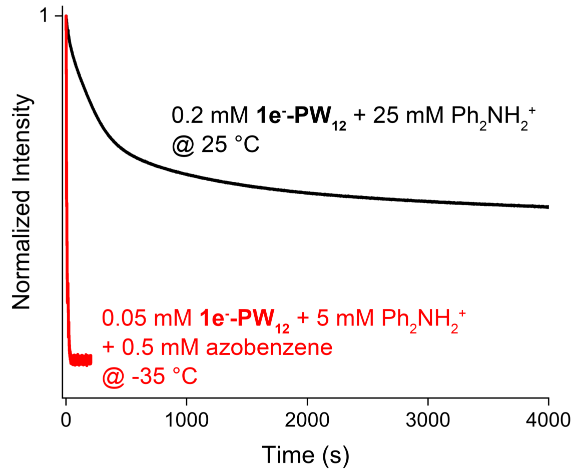

Figure S10. Normalized absorbance (λ= 765 nm, characteristic absorption of reduced **PW_{12}** cluster) changes over the time. The black curve indicates the H_2 evolution reaction by mixing 0.2 mM **$1e^-$-PW_{12}** cluster and 25 mM acid diphenylammonium tetrafluoroborate in MeCN at 25 °C; the red curve represents the semi-hydrogenation reaction of azobenzene by mixing 0.05 mM reduced **PW_{12}** cluster, 5 mM acid diphenylammonium tetrafluoroborate, and 0.5 mM azobenzene in MeCN at -35 °C. The comparison of the kinetics of these two reactions clearly indicates that the semi-hydrogenation reaction of azobenzene is significantly faster than the H_2 evolution reaction despite 4-time lower concentrations and 60-degree lower temperature.

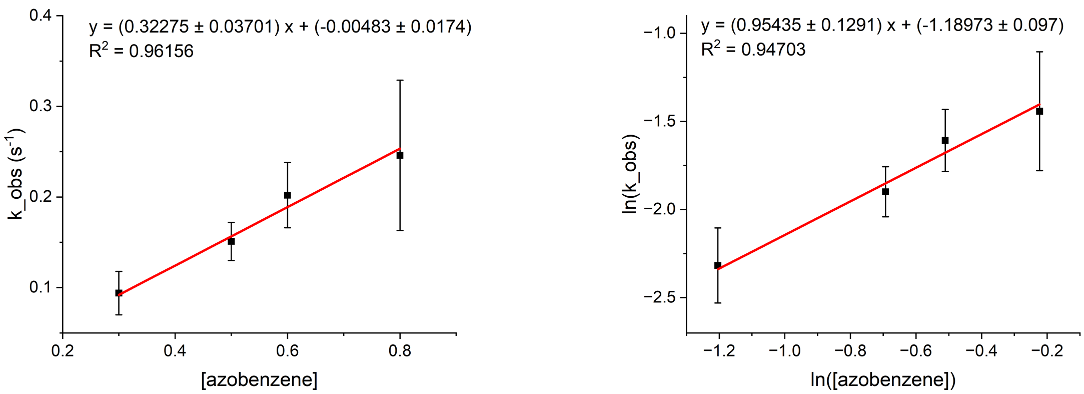

Figure S11. (Left) Plot of observed pseudo-1st-order rate constant (k_{obs}) as a function of [azobenzene] in MeCN at -35 °C. (Right) Plot of natural log of observed pseudo-1st-order rate constant (k_{obs}) as a function of natural log of [azobenzene] in MeCN at -35 °C. For the order of azobenzene, the concentration of [**1e$^-$-PW$_{12}$**] was kept constant at 0.05 mM and the acid diphenylammonium tetrafluoroborate ($Ph_2NH_2^+$) was introduced as 5 mM. Concentration of [azobenzene] was varied from 0.3 to 0.8 mM. The temperature was kept at -35 °C (238.15 K).

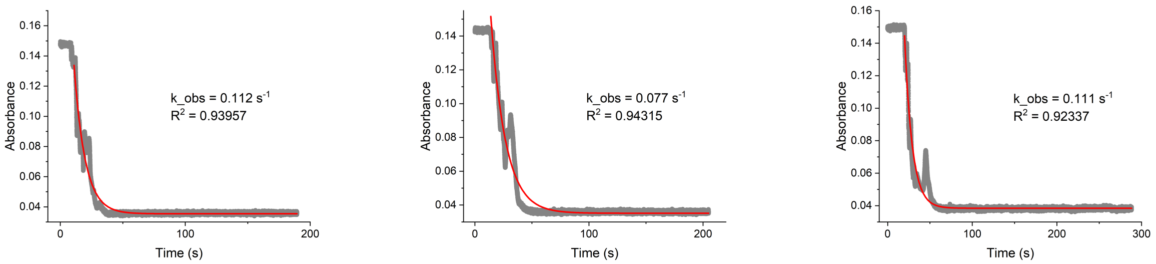

Figure S3. Plot of absorbance at 765 nm over time for reactions between azobenzene (0.3 mM), $Ph_2NH_2^+$ (5 mM), and **1e$^-$-PW$_{12}$** (0.05 mM) under pseudo-1st-order conditions recorded in MeCN at -35 °C with (grey) raw data and (red) fit curve, along with the fit-derived k_{obs} and R^2 parameters. Triplicate data sets are presented.

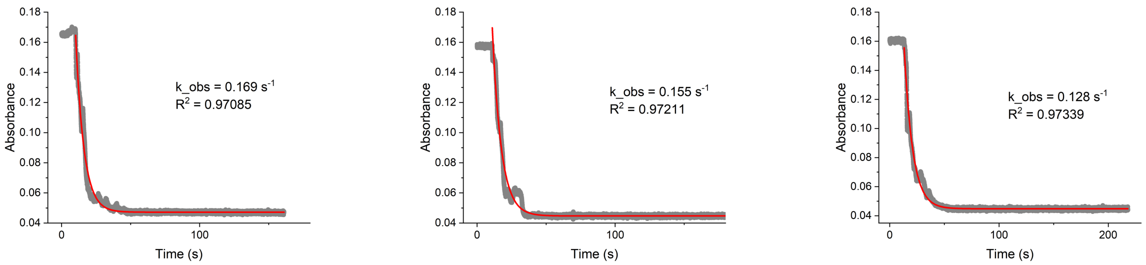

Figure S4. Plot of absorbance at 765 nm over time for reactions between azobenzene (0.5 mM), $Ph_2NH_2^+$ (5 mM), and **1e$^-$-PW$_{12}$** (0.05 mM) under pseudo-1st-order conditions recorded in MeCN at -35 °C with (grey) raw data and (red) fit curve, along with the fit-derived k_{obs} and R^2 parameters. Triplicate data sets are presented.

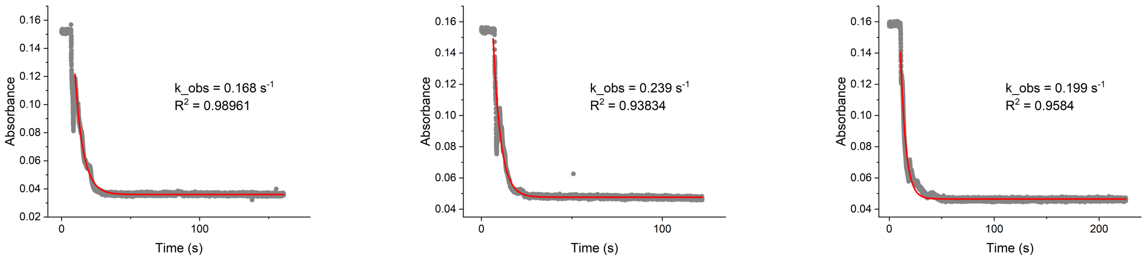

Figure S5. Plot of absorbance at 765 nm over time for reactions between azobenzene (0.6 mM), $Ph_2NH_2^+$ (5 mM), and **1e$^-$-PW$_{12}$** (0.05 mM) under pseudo-1st-order conditions recorded in MeCN at -35 °C with (grey) raw data and (red) fit curve, along with the fit-derived k_{obs} and R^2 parameters. Triplicate data sets are presented.

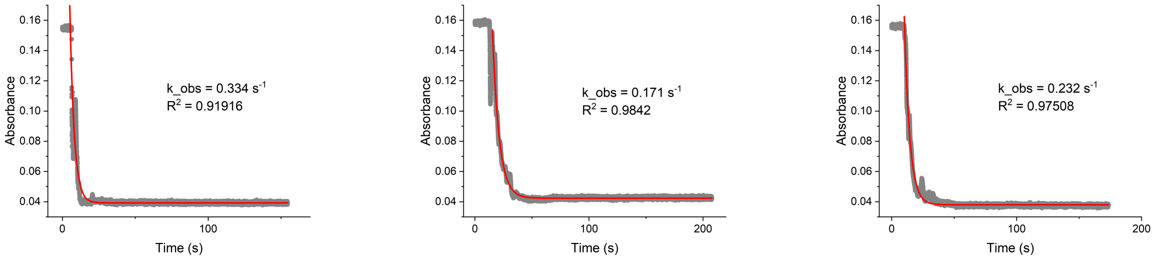

Figure S6. Plot of absorbance at 765 nm over time for reactions between azobenzene (0.8 mM), $Ph_2NH_2^+$ (5 mM), and **1e$^-$-PW$_{12}$** (0.05 mM) under pseudo-1st-order conditions recorded in MeCN at -35 °C with (grey) raw data and (red) fit curve, along with the fit-derived k_{obs} and R^2 parameters. Triplicate data sets are presented.

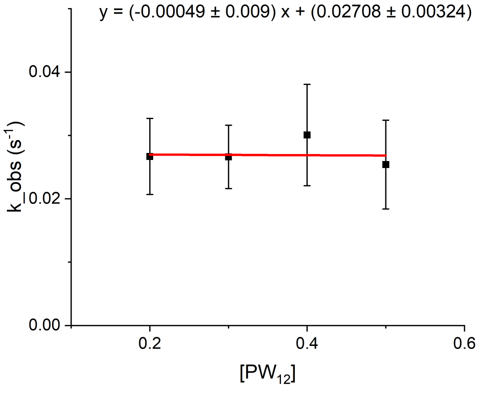

Figure S7. Plot of observed pseudo-1st-order rate constant (k_{obs}) as a function of [**1e$^-$-PW$_{12}$**] in MeCN at -35 °C. For the order of **1e$^-$-PW$_{12}$**, the concentration of azobenzene was kept constant at 5 mM and the acid diphenylammonium tetrafluoroborate ($Ph_2NH_2^+$) was introduced as 0.05 mM. Concentration of **1e$^-$-PW$_{12}$** was varied from 0.2 to 0.5 mM. The temperature was kept at -35 °C (238.15 K).

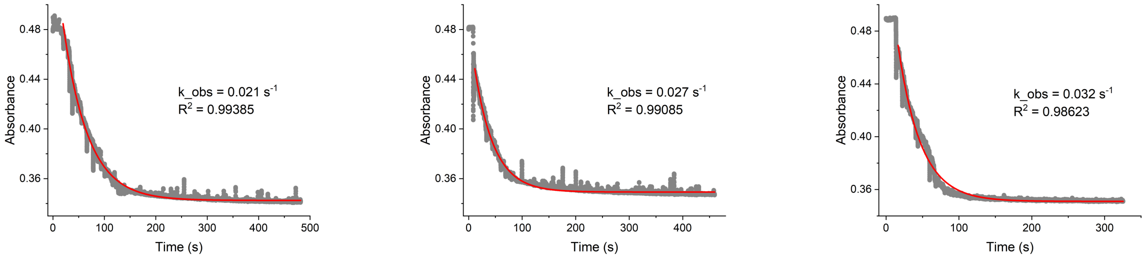

Figure S8. Plot of absorbance at 765 nm over time for reactions between **1e$^-$-PW$_{12}$** (0.2 mM), azobenzene (5 mM), and $Ph_2NH_2^+$ (0.05 mM) under pseudo-1st-order conditions recorded in MeCN at -35 °C with (grey) raw data and (red) fit curve, along with the fit-derived k_{obs} and R^2 parameters. Triplicate data sets are presented.

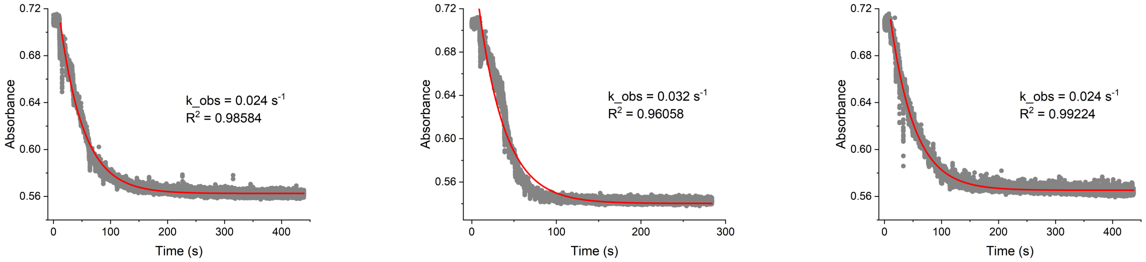

Figure S9. Plot of absorbance at 765 nm over time for reactions between **1e$^-$-PW$_{12}$** (0.3 mM), azobenzene (5 mM), and $Ph_2NH_2^+$ (0.05 mM) under pseudo-1st-order conditions recorded in MeCN at -35 °C with (grey) raw data and (red) fit curve, along with the fit-derived k_{obs} and R^2 parameters. Triplicate data sets are presented.

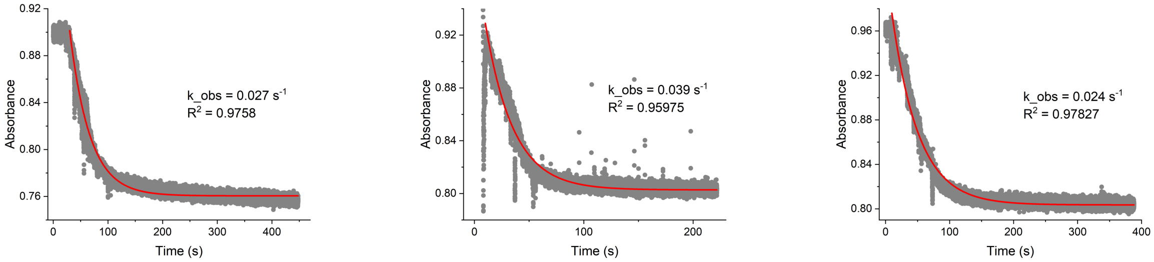

Figure S10. Plot of absorbance at 765 nm over time for reactions between **1e$^-$-PW$_{12}$** (0.4 mM), azobenzene (5 mM), and $Ph_2NH_2^+$ (0.05 mM) under pseudo-1st-order conditions recorded in MeCN at -35 °C with (grey) raw data and (red) fit curve, along with the fit-derived k_{obs} and R^2 parameters. Triplicate data sets are presented.

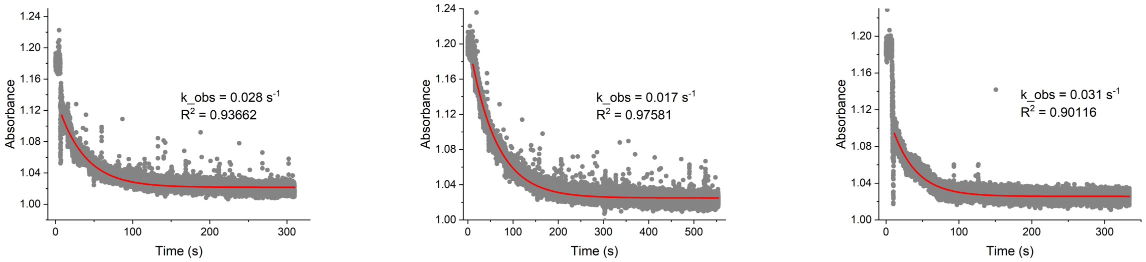

Figure S20. Plot of absorbance at 765 nm over time for reactions between **1e⁻-PW$_{12}$** (0.5 mM), azobenzene (5 mM), and $Ph_2NH_2^+$ (0.05 mM) under pseudo-1st-order conditions recorded in MeCN at -35 °C with (grey) raw data and (red) fit curve, along with the fit-derived k_{obs} and R^2 parameters. Triplicate data sets are presented.

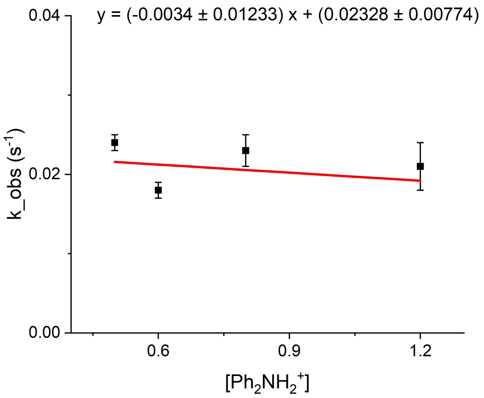

Figure S21. Plot of observed pseudo-1st-order rate constant (k_{obs}) as a function of [$Ph_2NH_2^+$] in MeCN at -35 °C. For the order of $Ph_2NH_2^+$, the concentration of azobenzene was kept constant at 5 mM and combined with 0.05 mM reduced cluster [**1e⁻-PW$_{12}$**]. Concentration of [$Ph_2NH_2^+$] was varied from 0.5 to 1.2 mM. The temperature was kept at -35 °C (238.15 K).

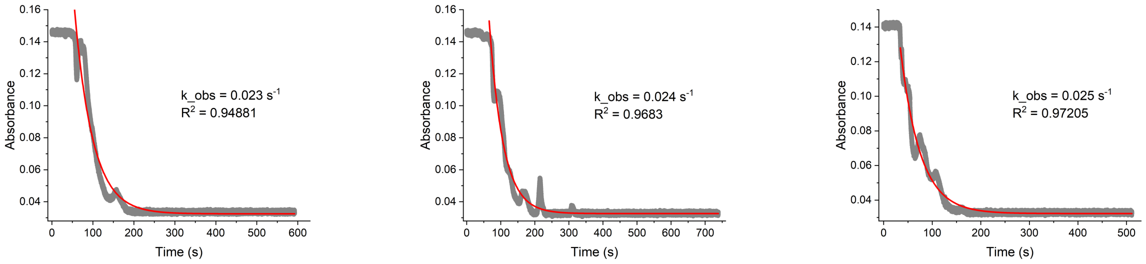

Figure S112. Plot of absorbance at 765 nm over time for reactions between $Ph_2NH_2^+$ (0.5 mM), azobenzene (5 mM), and **1e⁻-PW$_{12}$** (0.05 mM) under pseudo-1st-order conditions recorded in MeCN at -35 °C with (grey) raw data and (red) fit curve, along with the fit-derived k_{obs} and R^2 parameters. Triplicate data sets are presented.

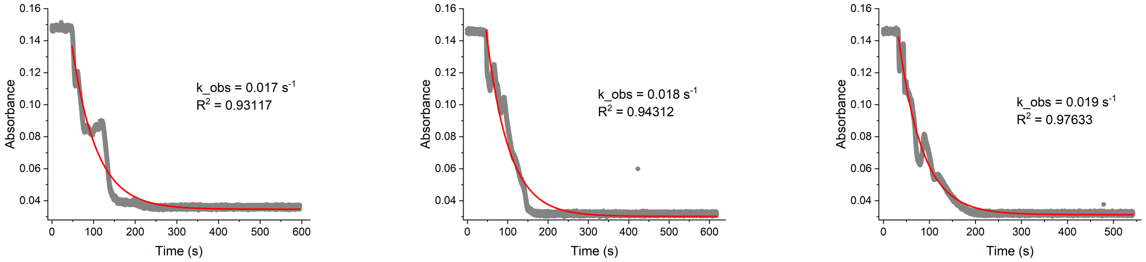

Figure S12. Plot of absorbance at 765 nm over time for reactions between $Ph_2NH_2^+$ (0.6 mM), azobenzene (5 mM), and **1e$^-$-PW$_{12}$** (0.05 mM) under pseudo-1st-order conditions recorded in MeCN at -35 °C with (grey) raw data and (red) fit curve, along with the fit-derived k_{obs} and R^2 parameters. Triplicate data sets are presented.

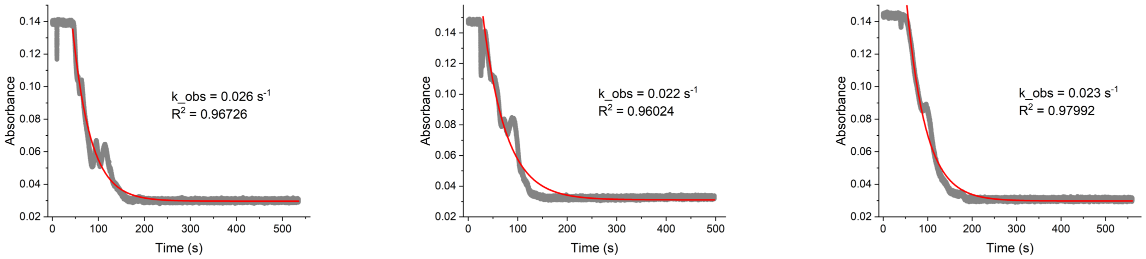

Figure S13. Plot of absorbance at 765 nm over time for reactions between $Ph_2NH_2^+$ (0.8 mM), azobenzene (5 mM), and **1e$^-$-PW$_{12}$** (0.05 mM) under pseudo-1st-order conditions recorded in MeCN at -35 °C with (grey) raw data and (red) fit curve, along with the fit-derived k_{obs} and R^2 parameters. Triplicate data sets are presented.

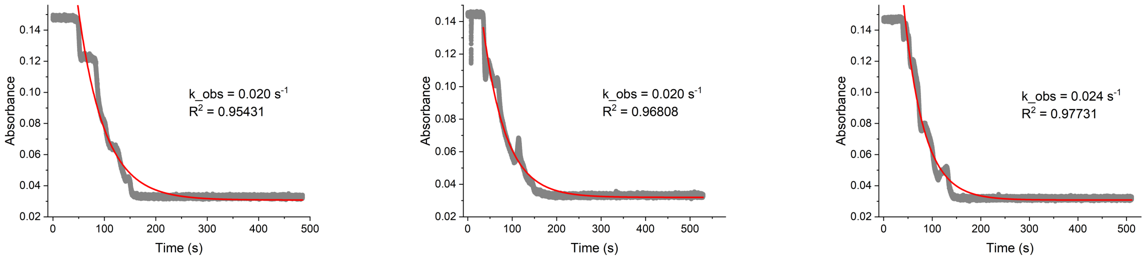

Figure S14. Plot of absorbance at 765 nm over time for reactions between $Ph_2NH_2^+$ (1.2 mM), azobenzene (5 mM), and **1e$^-$-PW$_{12}$** (0.05 mM) under pseudo-1st-order conditions recorded in MeCN at -35 °C with (grey) raw data and (red) fit curve, along with the fit-derived k_{obs} and R^2 parameters. Triplicate data sets are presented.

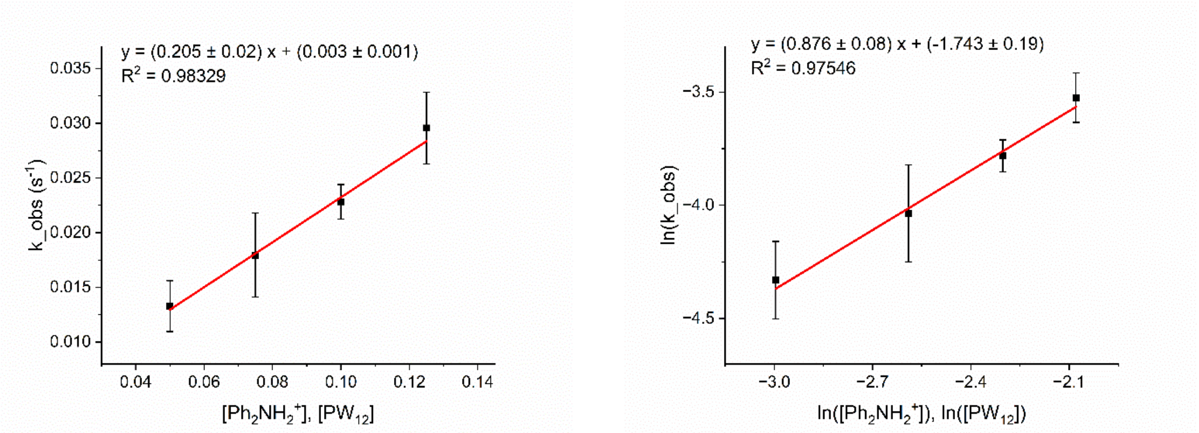

Figure S26. (Left) Plot of observed pseudo-1st-order rate constant (k_{obs}) as a function of $[Ph_2NH_2^+]$ or [**1e$^-$-PW$_{12}$**] (the concentration of *in situ* generated protonated, reduced $[PW_{12}O_{39}(OH)]^{3-}$) in MeCN at -35 °C. (Right) Plot of natural log of observed pseudo-1st-order rate constant (k_{obs}) as a function of natural log of $[Ph_2NH_2^+]$ or [**1e$^-$-PW$_{12}$**] (the concentration of *in situ* generated protonated, reduced $[PW_{12}O_{39}(OH)]^{3-}$) in MeCN at -35 °C. For the order of *in situ* generated $[PW_{12}O_{39}(OH)]^{3-}$, the concentration of [azobenzene] was kept constant at 5 mM. Concentrations of $[Ph_2NH_2^+]$ and [**1e$^-$-PW$_{12}$**] were synchronously varied from 0.05 to 0.125 mM. The temperature was kept at -35 °C (238.15 K).

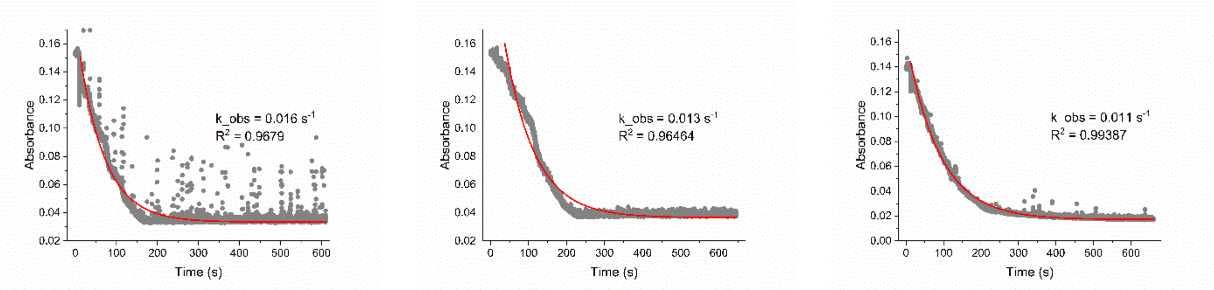

Figure S27. Plot of absorbance at 765 nm over time for reactions between azobenzene (5 mM), $Ph_2NH_2^+$ (0.05 mM), and **1e$^-$-PW$_{12}$** (0.05 mM) under pseudo-1st-order conditions recorded in MeCN at -35 °C with (grey) raw data and (red) fit curve, along with the fit-derived k_{obs} and R^2 parameters. Triplicate data sets are presented.

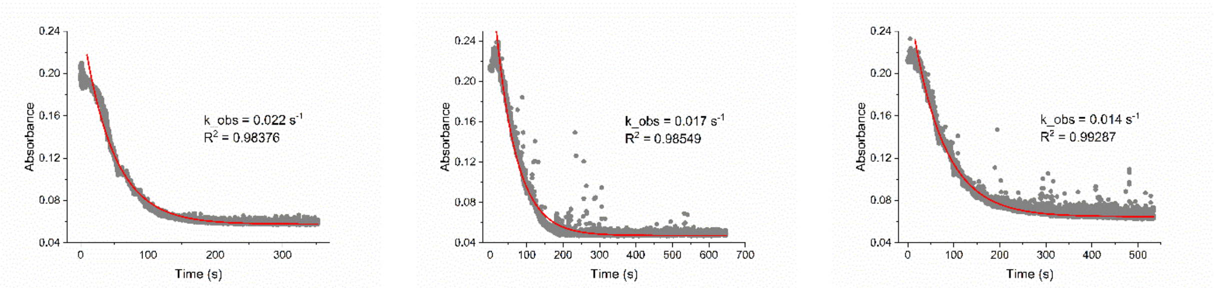

Figure S28. Plot of absorbance at 765 nm over time for reactions between azobenzene (5 mM), $Ph_2NH_2^+$ (0.075 mM), and **1e$^-$-PW$_{12}$** (0.075 mM) under pseudo-1st-order conditions recorded in MeCN at -35 °C with (grey) raw data and (red) fit curve, along with the fit-derived k_{obs} and R^2 parameters. Triplicate data sets are presented.

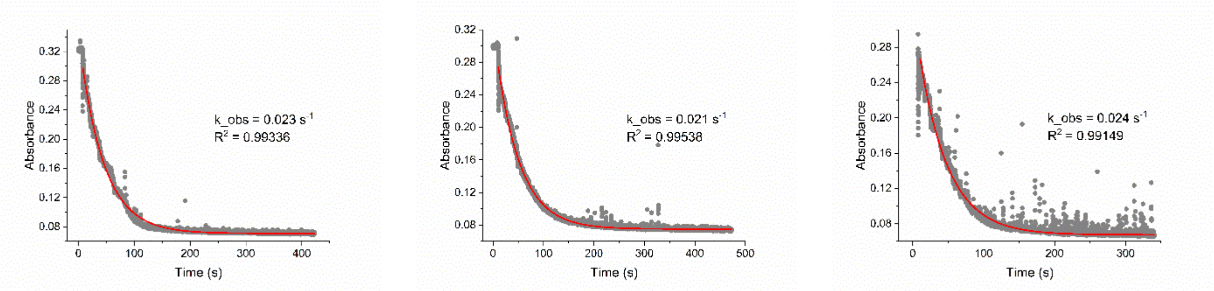

Figure S29. Plot of absorbance at 765 nm over time for reactions between azobenzene (5 mM), $Ph_2NH_2^+$ (0.1 mM), and **$1e^-$-PW_{12}** (0.1 mM) under pseudo-1st-order conditions recorded in MeCN at -35 °C with (grey) raw data and (red) fit curve, along with the fit-derived k_{obs} and R^2 parameters. Triplicate data sets are presented.

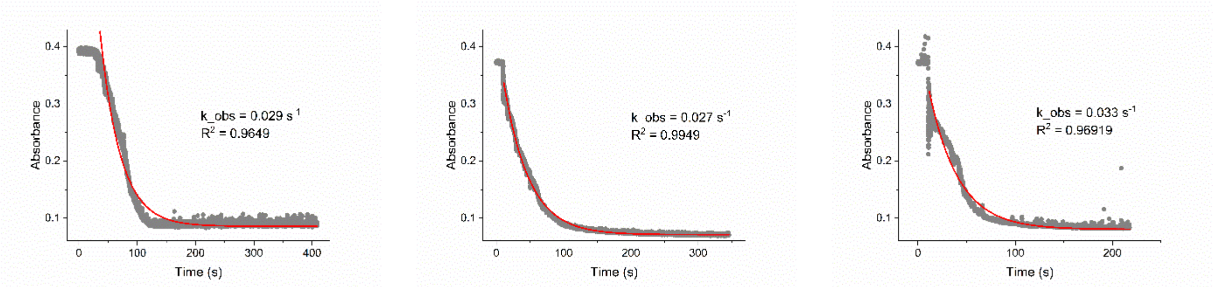

Figure S30. Plot of absorbance at 765 nm over time for reactions between azobenzene (5 mM), $Ph_2NH_2^+$ (0.125 mM), and **$1e^-$-PW_{12}** (0.125 mM) under pseudo-1st-order conditions recorded in MeCN at -35 °C with (grey) raw data and (red) fit curve, along with the fit-derived k_{obs} and R^2 parameters. Triplicate data sets are presented.

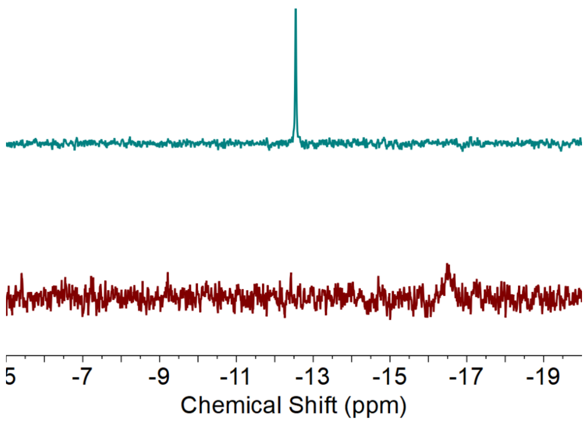

Figure S31. ^{31}P-NMR spectra of the mixture of **1e^{-}-PW$_{12}$**, azobenzene, and (top) weak acid benzoic acid (pKa(MeCN) = 21.5) or (bottom) strong acid diphenylammonium tetrafluoroborate ($Ph_2NH_2^+$, pKa(MeCN) = 5.98) at room temperature, 21 °C.

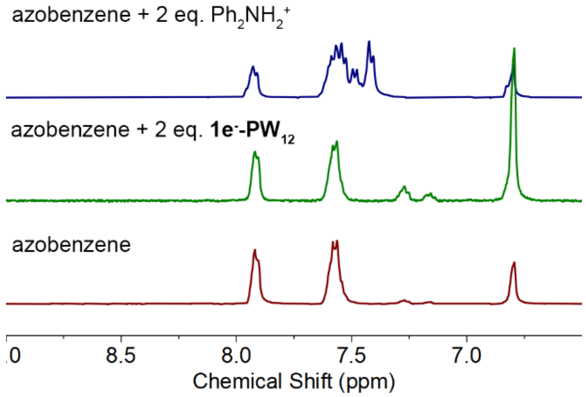

Figure S32. ^{1}H NMR spectra of (top) the mixture of 2 equivalents of $Ph_2NH_2^+$ and 1 equivalent of azobenzene, (middle) the mixture of 2 equivalents of **1e^{-}-PW$_{12}$** and 1 equivalent of azobenzene, and (bottom) azobenzene only in CD_3CN at room temperature, 21 °C.

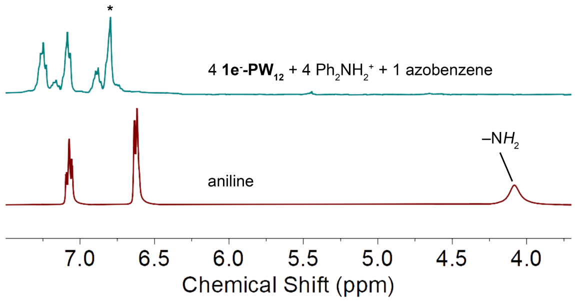

Figure S33. ^{1}H NMR spectra of (top) the mixture of 4 equivalents of **1e^{-}-PW$_{12}$** and $Ph_2NH_2^+$, 1 equivalent of azobenzene and (bottom) aniline only in CD_3CN at room temperature, 21 °C. The asterisk indicates the internal standard mesitylene. This result indicates that overdose of proton and electron does not further hydrogenate the hydrazobenzene to aniline.

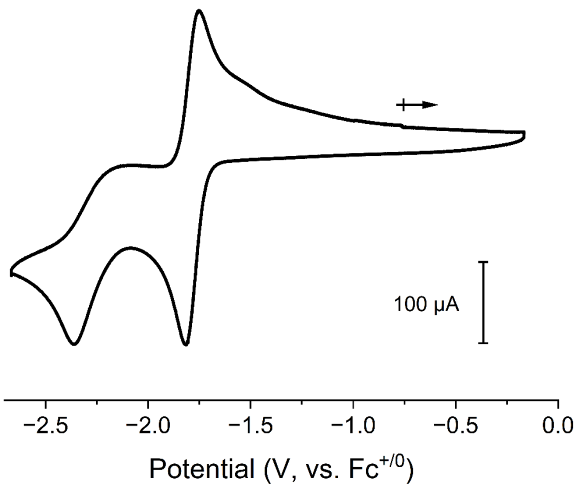

Figure S34. Cyclic voltammograms of 1 mM azobenzene obtained in acetonitrile with the scan rate of 100 mV/s, using 100 mM [^{n}Bu$_4$N]PF$_6$ as the supporting electrolyte. Ferrocene is used for measurement as the internal standard.

References

(1) Filowitz, M.; Ho, R. K. C.; Klemperer, W. G.; Shum, W. Oxygen-17 Nuclear Magnetic Resonance Spectroscopy of Polyoxometalates. 1. Sensitivity and Resolution. *Inorg. Chem.* **1979**, *18*, 93–103.
(2) Yu, H.-Z.; Yang, Y.-M.; Zhang, L.; Dang, Z.-M.; Hu, G.-H. Quantum-Chemical Predictions of pK_a's of Thiols in DMSO. *J. Phys. Chem. A* **2014**, *118*, 606–622.
(3) Bordwell, F. G.; Branca, J. C.; Bares, J. E.; Filler, R. Enhancement of the Equilibrium Acidities of Carbon Acids by Polyfluoroaryl Substituents. *J. Org. Chem.* **1988**, *53*, 780–782.
(4) Huffman, L. M.; Casitas, A.; Font, M.; Canta, M.; Costas, M.; Ribas, X.; Stahl, S. S. Observation and Mechanistic Study of Facile C-O Bond Formation between a Well-Defined Aryl–Copper(III) Complex and Oxygen Nucleophiles. *Chem. A Eur. J.* **2011**, *17*, 10643–10650.
(5) Maran, F.; Celadon, D.; Severin, M. G.; Vianello, E. Electrochemical Determination of the pK_a of Weak Acids in N,N-Dimethylformamide. *J. Am. Chem. Soc.* **1991**, *113*, 9320–9329.
(6) Kütt, A.; Tshepelevitsh, S.; Saame, J.; Lõkov, M.; Kaljurand, I.; Selberg, S.; Leito, I. Strengths of Acids in Acetonitrile. *Eur. J. Org. Chem.* **2021**, *2021*, 1407–1419.
(7) Tshepelevitsh, S.; Kütt, A.; Lõkov, M.; Kaljurand, I.; Saame, J.; Heering, A.; Plieger, P. G.; Vianello, R.; Leito, I. On the Basicity of Organic Bases in Different Media. *Eur. J. Org. Chem.* **2019**, *2019*, 6735–6748.
(8) Vallaro, M.; Ermondi, G.; Saame, J.; Leito, I.; Caron, G. Ionization and Lipophilicity in Nonpolar Media Mimicking the Cell Membrane Interior. *Bioorg. Med. Chem.* **2023**, *81*, 117203.
(9) Bordwell, F. G. Equilibrium Acidities in Dimethyl Sulfoxide Solution. *Acc. Chem. Res.* **1988**, *21*, 456–463.
